# Supplementary figures and images for: Porcine Placenta Peptide Inhibits UVB-Induced Skin Wrinkle Formation and Dehydration: Insights into MAPK Signaling Pathways from In Vitro and In Vivo Studies
Source: Int J Mol Sci. 2023 Dec 20;25(1):83. doi: 10.3390/ijms25010083 (PMC10778591; doi:10.3390/ijms25010083)

Figure S1.

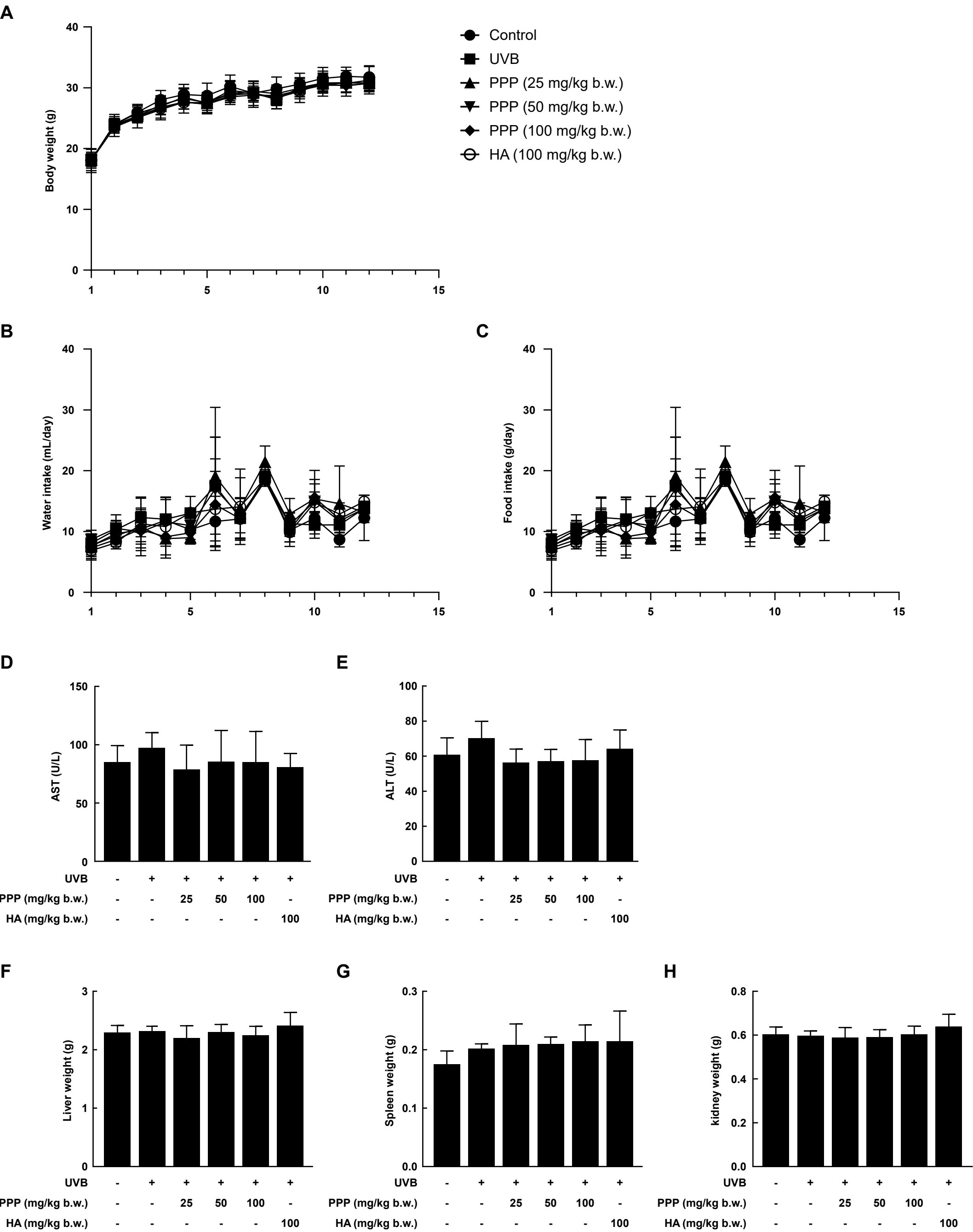

Supplement: Supplementary file 1 [file ijms-25-00083-s001.zip › ijms-2706335-supplementary.pdf]
